# Concurrent chronic myeloid leukemia and *CALR*-mutated myeloproliferative neoplasm

**DOI:** 10.17179/excli2019-2063

**Published:** 2020-01-08

**Authors:** Stephen E. Langabeer

**Affiliations:** 1 Cancer Molecular Diagnostics, St. James's Hospital, Dublin, Ireland

## ⁯

***Dear Editor,***

After the *JAK2* V617F mutation, insertion and/or deletion (indel) mutations of *CALR* exon 9 are the second most common driver mutations in the myeloproliferative neoplasms (MPN) of essential thrombocythemia and primary myelofibrosis and their detection is considered a major diagnostic criterion for these malignancies. It is becoming increasingly apparent that MPNs harboring *CALR* mutations (along with the mutations of *JAK2* V617F and *MPL* exon 10) may occur in patients with *BCR-ABL1*-positive chronic myeloid leukemia (CML) as evidenced by a wave of recently reported cases. The *CALR*-positive MPN and CML may appear concurrently with composite morphology or sequentially with either malignancy revealed as a consequence of specific treatment for one of the malignancies (Table 1(Tab. 1); References in Table 1: Balducci et al., 2019[1]; Blouet et al., 2018[2]; Boddu et al., 2018[3]; Bonzheim et al., 2015[4]; Cabagnols et al., 2015[5]; da Costa et al., 2019[6]; De Roeck et al., 2018[7]; Diamond et al., 2016[8]; Dogliotti et al., 2017[9]; Gilles et al., 2015[10]; Guidotti et al., 2020[11]; Jeromin et al., 2017[12]; Kandarpa et al., 2017[13]; Klairmont et al., 2018[14]; Lewandowski et al, 2018[15]; Loghavi et al., 2015[16]; Nomani et al., 2016[18]; Pagoni et al., 2014[19]; Seghatoleslami et al, 2016[20]; Xia et al., 2019[21]). Review of patients shows that the presenting malignancy was unknown in one case, CML in 11/24 (46 %) and *CALR*-mutated MPN in the remaining 12/24 (50 %) cases. Evidence exists for molecular abnormalities occurring within a single clone and in distinct clonal populations.

While co-existence of CML and another MPN has clinical relevance with respect to selection and timing of tyrosine kinase inhibitor therapy, there is currently insufficient follow-up data to ascertain overall survival of such cases. There is limited value in assessing the *JAK2 *V617F mutation in all newly presenting CML cases (McCarron et al., 2012[17]): screening for the less frequent *CALR* and *MPL* mutations in all likelihood would show a similar redundancy. Given the low incidence but increasing awareness of co-existing CML and MPN, testing for the relevant rearrangement should therefore be implemented when there is clinical, hematological or morphological evidence.

## Conflict of interest

The author declares no conflicts of interest.

## Figures and Tables

**Table 1:**
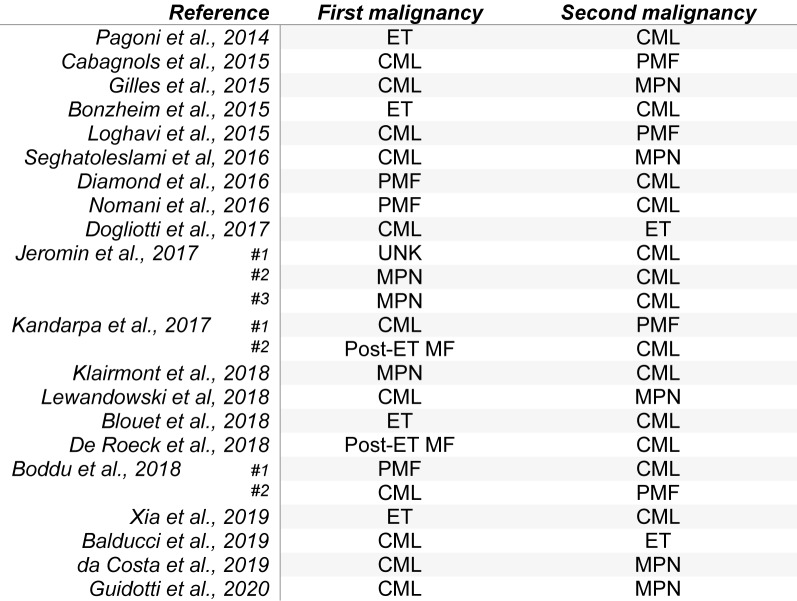
Clinical presentation order of cases of co-existing *BCR-ABL1*-positive chronic myeloid leukemia (CML) and *CALR*-positive myeloproliferative neoplasm (MPN). ET: essential thrombocythemia; PMF: primary myelofibrosis; MF: myelofibrosis; UNK: unknown
